# Elektrokonvulsionstherapie: Was tun, wenn der Bedarf die Behandlungskapazitäten übersteigt? – Eine medizinethische Handlungsempfehlung

**DOI:** 10.1007/s00115-024-01663-z

**Published:** 2024-04-15

**Authors:** David Zilles-Wegner, Matthias Besse, Isabel Methfessel, Alfred Simon

**Affiliations:** 1 Klinik für Psychiatrie und Psychotherapie, Universitätsmedizin Göttingen, Von-Siebold-Str. 5, 37075 Göttingen, Deutschland; 2 Akademie für Ethik in der Medizin, Göttingen, Deutschland

## Hintergrund

Die Elektrokonvulsionstherapie (EKT) ist ein evidenzbasiertes und in Leitlinien empfohlenes Therapieverfahren, das insbesondere bei schwer ausgeprägten oder pharmakotherapieresistenten affektiven, psychotischen und katatonen Syndromen indiziert ist [1]. Die regionale Verfügbarkeit und damit der Zugang von Patienten zur EKT ist in Deutschland sowie international sehr heterogen. Trotz des für die letzten Dekaden in Deutschland dokumentierten deutlichen Anstiegs der Behandlungszahlen ist der Anteil der psychiatrischen Kliniken mit EKT-Angebot bei knapp 50 % gleichgeblieben [2, 3]. Mit der fehlenden regionalen Verfügbarkeit geht eine ebenfalls zunehmende Rate von durchschnittlich 18 % externen Zuweisungen zur EKT einher [3], die zu praktischen Problemen in der Versorgung führen kann [4]. Wenngleich sich diese unzureichende Versorgung vielerorts in der klinischen Praxis zeigt, fehlen dazu bislang systematisch erhobene Daten. Die Quantifizierung und Identifikation von Implementierungshürden ist Ziel aktuell in Planung befindlicher Studien der Versorgungsforschung.

## Konsequenzen fehlender EKT‑Ressourcen

Die Konsequenzen fehlender oder eingeschränkt verfügbarer EKT wurden im Kontext der Corona-Pandemie deutlich [5, 6]. Mehrere Zentren publizierten unabhängig voneinander Daten, die konsistent hohe Rezidiv- und Rehospitalisierungsraten bei Patienten mit reduzierter oder ausgesetzter Erhaltungs-EKT zeigten [7, 8]. Aus methodischen Gründen schwieriger systematisch zu untersuchen sind die Auswirkungen, wenn indizierte Akutbehandlungen aufgrund fehlender Ressourcen nicht oder nur mit großer zeitlicher Verzögerung stattfinden. Kasuistiken beschreiben diese jedoch eindrücklich, wenn etwa ein Patient mit therapieresistenter Katatonie erst nach 148 Tagen unter intensivmedizinischer, supportiver Behandlung in eine Klinik mit EKT verlegt werden konnte, in der die katatone Symptomatik dann im Verlauf weniger Behandlungen remittierte [9]. Generell ist die zeitnahe Bereitstellung einer Behandlungsmöglichkeit angezeigt, da eine längere Episodendauer mit einem schlechteren Ansprechen auf EKT wie auch andere psychiatrische Therapieverfahren einhergeht. In einer internationalen Studie zu den Auswirkungen der Pandemie auf die Versorgung mit EKT wurden Suizide und andere Todesfälle beschrieben, die kausal dem fehlenden Zugang zur EKT zugeschrieben wurden [6]. Bereits während der Pandemie wurden klinikinterne Strategien entwickelt, um Behandlungen angesichts zu geringer Ressourcen zu priorisieren. Zum Teil erfolgte dies in individueller Abstimmung und Nutzen-Risiko-Abwägung gemeinsam mit den Patienten [7], zum Teil mittels operationalisierter Skalen zur Risikoeinschätzung [10].

## Bereitstellung von Infrastruktur und Personal

Die verfügbaren Behandlungskapazitäten zur EKT sind nach Kenntnis der Autoren an vielen Kliniken (unabhängig von temporär zusätzlichen Einschränkungen) nicht ausreichend, um alle Patienten mit bestehendem Bedarf adäquat und zeitgerecht zu versorgen. Entsprechende Anfragen an das Referat Hirnstimulationsverfahren, Sektion Elektrokonvulsionstherapie der Deutschen Gesellschaft für Psychiatrie und Psychotherapie, Psychosomatik und Nervenheilkunde (DGPPN) e. V. haben zu einem Positionspapier der DGPPN sowie der Arbeitsgemeinschaft für Neuropsychopharmakologie und Pharmakopsychiatrie (AGNP) geführt [11]. In diesem unterstützen die Fachgesellschaften die Forderung der Kliniken nach einer indikationsgerechten Bereitstellung der für die EKT notwendigen Infrastruktur sowie personellen Ausstattung inklusive der erforderlichen anästhesiologischen Ressourcen. Eine als indiziert festgestellte EKT muss durchgeführt werden können; ein Ausfall aufgrund mangelnder Ressourcen ist aus wissenschaftlich-medizinischen sowie medizinethischen Gründen [12, 13] inakzeptabel. Solange die entsprechenden Ressourcen nicht bedarfsgerecht vorhanden sind, werden Kliniken jedoch mit dem Missverhältnis aus Indikationshäufigkeit und verfügbaren Behandlungen umgehen müssen.

## Priorisierungserfordernis bei nicht ausreichenden Behandlungskapazitäten

Allgemein bilden Indikation und Patientenwille die Grundlage jeder patientenzentrierten Therapieentscheidung. Beide sind vor Aufnahme einer EKT zu evaluieren sowie im weiteren Verlauf der Therapie regelmäßig zu reevaluieren. Wurde eine EKT initial gegen den natürlichen Patientenwillen begonnen, so ist im weiteren Verlauf der Therapie eine Zustimmung anzustreben.

Eine EKT ist zu unterlassen bzw. zu beenden,

- wenn diese nicht indiziert ist, weil
  - ein Ansprechen auf die Therapie nicht zu erwarten ist,
  - ein adäquater Therapieversuch sich als wirkungslos erwiesen hat,
  - eine Kontraindikation besteht,
- bei entgegenstehendem autonomem Patientenwillen (aktuell, vorausverfügt, zuvor mündlich geäußert oder mutmaßlich).

In allen anderen Fällen sollte die EKT als Behandlungsoption zur Verfügung gestellt werden. Wenn der Bedarf an EKT die Behandlungskapazitäten übersteigt, ist eine Priorisierung erforderlich. Aus klinischer Sicht stellt sich vor allem die Frage, nach welchen Kriterien eine ggf. notwendige Priorisierung von Behandlungen erfolgen sollte (s. Tab. 1).

**Tab. 1:** Potenzielle Kriterien einer Priorisierungsentscheidung

Dringlichkeit der Indikation
Konsequenzen bei Nichtbehandlung
Wartezeit
Erfolgsaussichten
Regionale Zuständigkeit der Klinik

An der Klinik für Psychiatrie und Psychotherapie der Universitätsmedizin Göttingen wurden diese Kriterien gemeinsam mit der Akademie für Ethik in der Medizin interdisziplinär auf der Grundlage der medizinethischen Prinzipien Wohltun, Nicht-Schaden, Respekt der Selbstbestimmung und Gerechtigkeit [14] analysiert. Da es zu dem Thema der Priorisierung von EKT in Deutschland noch keine spezifischen rechtlichen Regelungen, Leitlinien oder Empfehlungen gibt, wurden bei der Erstellung der nachfolgenden Handlungsempfehlungen andere vergleichbare rechtlichen Vorgaben (insbesondere aus dem Transplantations- sowie dem Infektionsschutzgesetz), die klinisch-ethischen Empfehlungen zur Zuteilung intensivmedizinischer Ressourcen im Kontext der COVID-19-Pandemie [15], die Empfehlungen der Schweizerischen Akademie der Medizinischen Wissenschaften zur außerordentlichen Ressourcenknappheit in der stationären Versorgung [16] sowie ein EKT-spezifischer internationaler Vorschlag [10] berücksichtigt.

## Handlungsempfehlung zur Priorisierung von Elektrokonvulsionstherapie

Eine Priorisierung bzw. Posteriorisierung aufgrund von Kriterien wie sozialer Status, Alter, Geschlecht, Nationalität, Religion, Versichertenstatus oder Behinderung ist nicht zulässig. Übersteigt die Zahl der Anfragen für eine EKT die vorhandenen Behandlungskapazitäten, ist zu prüfen, ob die lokale Ressourcenknappheit durch eine angemessene Allokation behoben werden kann (z. B. Fokussierung auf die Behandlung von Patienten aus dem eigenen Einzugsbereich, Verlegung von Patienten in andere Kliniken mit freien Behandlungskapazitäten).

Lässt sich die lokale Ressourcenknappheit nicht durch Allokation beheben, muss zunächst eine *Priorisierung nach Dringlichkeit* erfolgen. Es sollen also zunächst Behandlungen aufgeschoben werden, bei denen durch die zeitliche Verzögerung keine Verschlechterung der Prognose, keine irreversiblen Gesundheitsschädigungen oder kein vorzeitiger Tod zu erwarten sind.

Bei gleicher Dringlichkeit erfolgt eine *Priorisierung nach Wartezeit*, d. h. solche Patienten werden vorrangig behandelt, die bereits längere Zeit auf eine EKT warten.

Für den sehr unwahrscheinlichen Fall, dass die vorhandenen Behandlungskapazitäten nicht ausreichen, um alle akut lebensbedrohlich erkrankten Patienten zu behandeln, sollten vorrangig diejenigen Patienten mit EKT behandelt werden, bei denen eine höhere Wahrscheinlichkeit besteht, dass die Therapie anspricht [17] und der lebensbedrohliche Zustand damit abgewendet werden kann. In allen anderen Fällen ist das *Kriterium der Erfolgsaussicht* bereits damit erfüllt, dass im Rahmen der Indikationsstellung festgestellt worden ist, dass eine begründete Aussicht auf Therapieerfolg besteht (Stichwort: „Minimalnutzenschwelle“ [18, Rn. 45]). Diese auf medizinischer Evidenz und klinischer Erfahrung beruhende Einschätzung ist im weiteren Verlauf der Therapie regelmäßig zu reevaluieren.

Die besondere Verantwortung der Klinik gegenüber Patienten aus dem eigenen Einzugsgebiet schließt nicht aus, dass auch Patienten aus dem Einzugsbereich anderer Kliniken auf entsprechende Anfrage behandelt werden, sofern diese nicht an der für sie zuständigen Klinik behandelt werden können. Eine Priorisierung von Patienten aus anderen Einzugsbereichen gegenüber Patienten aus dem eigenen Einzugsbereich kann erfolgen, wenn eine sehr hohe Dringlichkeit besteht.

Kommt es aufgrund nicht ausreichender Behandlungskapazitäten zu einer Verschiebung einer EKT, so sollte der Patient bzw. der rechtliche Vertreter sowie ggf. die Angehörigen über die Gründe der Verschiebung und die damit möglicherweise verbundenen gesundheitlichen Auswirkungen transparent informiert werden. Ferner sollte versucht werden, den Patienten bestmöglich alternativ zu behandeln. Bei ethischen Konflikten kann ein Klinisches Ethikkomitee beratend hinzugezogen werden.

## Fazit für die Praxis

- Verfügbare Daten deuten darauf hin, dass aktuell keine ausreichenden Ressourcen bestehen, EKT flächendeckend bedarfsgerecht anzubieten.
- Findet eine indizierte EKT oder Erhaltungs-EKT nicht statt, kann dies zu protrahierten Krankheitsverläufen sowie Rezidiven bis hin zu Todesfällen führen.
- Notwendige Priorisierungen sollten medizinisch und medizinethisch begründet sein.
- Primäres Kriterium ist dabei die Dringlichkeit der Indikation, d. h. die Gefahr einer irreversiblen Gesundheitsschädigung oder Verschlechterung der Prognose.
